# Factors associated with and patterns of alcohol intake during the first year of breast cancer survivorship

**DOI:** 10.1007/s11764-025-01893-3

**Published:** 2025-09-24

**Authors:** Sanjna Rajput, Robert A. Vierkant, Nicole L. Larson, Daniela L. Stan, Dawn M. Mussallem, Stacy D. D’Andre, Fergus J. Couch, Janet E. Olson, Ciara C. O’Sullivan, Kathryn J. Ruddy

**Affiliations:** 1 Mayo Clinic School of Graduate Medical Education, Internal Medicine Residency, Mayo Clinic, Rochester, MN USA; 2 Department of Quantitative Health Sciences, Mayo Clinic, Rochester, MN USA; 3 Department of General Internal Medicine, Mayo Clinic, Rochester, MN USA; 4 Department of General Internal Medicine, Mayo Clinic, Jacksonville, FL USA; 5 Department of Oncology, Mayo Clinic, 200 First Street SW, Rochester, MN 55905 USA; 6 Department of Laboratory Medicine and Pathology, Mayo Clinic, Rochester, MN USA

**Keywords:** Aclohol consumption, Breast cancer survivorship, Health behaviors, Lifestyle factors, Cancer risk reduction

## Abstract

**Purpose:**

To evaluate alcohol intake patterns, and identify factors associated with alcohol use in early survivorship among breast cancer survivors, as well as associations with changes in alcohol consumption in early survivorship.

**Methods:**

Patients diagnosed with breast cancer (stages 0–3) between 2014 and 2022 were surveyed regarding their average alcohol intake near the time of diagnosis and at approximately one year post-diagnosis. Alcohol intake was categorized into four levels and associations with demographic, clinical, and behavioral factors were assessed using bivariable and multivariable models. Changes in alcohol intake over time were examined using McNemar’s and Bowker’s tests and multinomial logistic regression.

**Results:**

In this cohort of 2080 participants, the proportion of participants consuming at least one drink per week decreased from 71.2% at diagnosis to 39.8% at one year post-diagnosis, while the proportion reporting less than one drink per week increased from 28.9 to 60.2%. Reduction in alcohol intake at one year post-diagnosis was more likely among those experiencing financial difficulty, receiving chemotherapy, reporting lower physical health, and current or never smokers compared to former smokers. Better physical health, engagement in moderate-intensity exercise, and current smoking were associated with higher cross-sectional alcohol intake at one year post-diagnosis.

**Conclusions:**

Alcohol consumption declined significantly in early survivorship. Financial, behavioral, and treatment-related factors played key roles in shaping these patterns.

**Implications for Cancer Survivors:**

Our findings underscore the importance of targeted interventions to support personalized counseling on alcohol intake, particularly for individuals with better physical health, greater financial stability, or co-occurring risky behaviors like smoking.

**Supplementary Information:**

The online version contains supplementary material available at https://doi.org/10.1007/s11764-025-01893-3.

## Introduction

Breast cancer is the most prevalent cancer among women, with 310,720 cases of invasive breast cancer and 56,500 cases of ductal carcinoma in situ (DCIS) diagnosed in the USA in 2024 [1]. As of January 2022, there were 4 million breast cancer survivors in the USA, accounting for 22% of cancer survivors nationally. Due to advances in early detection and treatment of breast cancer, as well as an aging population, the population of breast cancer survivors is expected to continue to grow [2]. This expanding survivor population heightens the importance of addressing modifiable lifestyle factors that may impact prognosis, quality of life, and long-term outcomes in survivorship care.

Among these modifiable factors, alcohol intake has garnered significant attention due to its established role in breast cancer incidence and prognosis [3–8]. Alcohol intake, both prior to and after a diagnosis of breast cancer, has been associated with poorer breast cancer outcomes, including increased risk of recurrence and breast cancer-specific mortality [9, 10]. There seems to be a modest association between the risk of recurrence and consuming more than two units of alcohol per day (i.e., more than 24 g of alcohol per day) before a breast cancer diagnosis, as well as with a history of > 40 drinking years (with a “drinking year” defined as one unit of alcohol per day in one year from age 20 until one year before the time of diagnosis) [11]. Moreover, post-diagnosis alcohol intake appears to have stronger associations with adverse outcomes. One study found that consuming more than one alcoholic drink (> 12 g of alcohol per day) after diagnosis increased the risk of late recurrence at 5 years post-diagnosis by 28% compared to not drinking or consuming 0.36–6 g or > 6– < 12 g of alcohol per day [12], and another study observed a dose-dependent increase in recurrence risk [13]. Kwan et al. reported that postmenopausal women consuming six or more grams of alcohol daily faced a 51% higher risk of recurrence and a 72% higher risk of mortality, with overweight and obese women most affected [10, 14]. Moreover, the health impacts of alcohol intake during survivorship extend beyond cancer-specific outcomes, influencing treatment-related adverse effects such as cognition and cardiotoxicity [15, 16].

Current survivorship guidelines recommend that survivors either abstain from alcohol or limit consumption to no more than one standard drink per day [17]. However, adherence to these recommendations may be suboptimal, with some breast cancer survivors consuming alcohol at levels comparable to or exceeding those of the general population [18]. Importantly, alcohol intake patterns are not static, and individuals may modify their drinking habits following a cancer diagnosis [19]. The factors driving these changes are not well understood but may include demographic factors such as younger age at diagnosis and financial stability, psychosocial factors such as current or former smoking and physical health status, and treatment-related variables such as receipt of chemotherapy [18, 20]. The first year following cancer diagnosis represents a critical window within early survivorship as a period of behavior adjustment often referred to as a “teachable moment.” This period, marked by disruption and reflection following the cancer experience, may heighten survivors’ receptivity to adopting healthier behaviors as a way to feel a sense of control over their risk of recurrence [21]. Understanding early trajectories in alcohol intake post-diagnosis will help inform survivorship care and health policy interventions in at-risk groups.

Our group previously identified that younger age, White race, financial security, mild to moderate intensity exercise, and smoking are associated with more alcohol consumption prior to a breast cancer diagnosis [22]. Here, we assessed changes in alcohol intake over the year following a breast cancer diagnosis, investigating demographic, lifestyle, and clinical characteristics that were associated with increasing or decreasing alcohol intake over time.

## Methods

Patients seen at least once at the Mayo Clinic in Rochester, Minnesota, who provided informed consent are prospectively enrolled in the Mayo Clinic Breast Disease Registry (MCBDR), following an initial diagnosis of breast cancer within the year prior (at age 18 or older). Soon after enrollment, a baseline survey (baseline) was mailed between 2015 and 2022 to 4440 prospective MCBDR participants who were diagnosed with breast cancer between 2014 and 2022, with another survey mailed approximately 1 year after breast cancer diagnosis (year 1).

Participants were included in this analysis if they responded to the alcohol intake question on both the baseline and year 1 surveys. Participants were excluded if they had stage IV disease, breast cancer recurrence at any time prior to the return of the year 1 survey, or if their baseline survey was returned more than 12 months after diagnosis. Participants with other stages (0–3 and missing) were included in the dataset.

The baseline survey included an item asking about the average number of alcoholic drinks consumed per week over the past 10 years. The year 1 survey included an item asking about the average number of alcoholic drinks consumed per week over the past year. In both surveys, this was specified as 1 standard drink = 5 oz of wine, 12 oz of beer, or 1 oz of liquor. Self-reported alcohol intake was classified into 4 categories of alcohol intake per week: none or less than < 1, 1–4 (low), 5–14 (moderate), and 15 + (high). Patients were also asked about demographic and clinical characteristics including education level (high school graduate or less, vocational education beyond high school, college or associate’s degree, Bachelor’s degree, Graduate school), financial situation (difficulty paying bills or little spare money), smoking status (never, former, or current), exercise levels (minutes of mild-intensity, moderate-intensity, and strenuous-intensity exercise per week), menopausal status (pre-menopausal or post-menopausal), and PROMIS-10 scores. The PROMIS Global-10 is a 10-item measure of health, well-being, and distress that produces global physical health (GPH) and global mental health (GMH) scores. PROMIS measures use a *T*-score metric that is converted from raw sum scores. The *T*-score of 50 is standardized to the mean US general population with a standard deviation (SD) of 10 points [23]. A higher *T*-score demonstrates more of what is being measured, such as greater global physical or mental health than the general population. Nurse-abstracted data on race, gender, age at cancer diagnosis, tumor stage, tumor characteristics, and cancer treatments received after diagnosis (surgery, radiation, chemotherapy, and/or endocrine therapy), and *BRCA* mutation status were collected from the electronic health record and used for this analysis.

Data were summarized using frequencies and percentages for categorical variables and means and standard deviations for continuous variables. Bivariable cross-sectional associations of year 1 alcohol intake with demographic and clinical characteristics, and with PROMIS-10 scores, were examined using Monte Carlo-based Fisher exact tests. In brief, for a given comparison, a cross-tabulated table was created using the observed data and the Fisher test statistic was extracted. Following this, 10,000 random samples of cross-tabulated tables were generated, conditional on the same total sample size, row totals, and column totals as the observed table. The Monte Carlo *p*-value was then calculated as the proportion of the resulting 10,000 tables that yielded a test statistic at least as large as the one observed. We chose Monte Carlo-based tests rather than traditional exact tests because the number of permutations needed to calculate the exact tests was extremely large and memory intensive. Following these bivariable associations, we fit a nominal, multivariable, multi-categorical logistic regression model to determine variables independently associated with year 1 alcohol intake, after accounting for the effects of all other bivariably significant characteristics. Alcohol intake was fit as the outcome, and all variables that were associated with alcohol intake (*p* < 0.05) in the bivariable models were included in the multivariable model, using a multinomial distribution and a generalized logit (glogit) link function. Because multinomial logistic regression odds ratios can be challenging to interpret, we instead presented estimated marginal percentages of patients falling within a given alcohol intake category for each level of each characteristic of interest, using an approach similar to calculating least squares means when using a continuous outcome variable. First, predicted probabilities of alcohol intake were calculated for every combination of variables in the model. Then, for a given category of a given characteristic, we calculated the mean of the predicted probabilities across all participants falling in that category. These marginal predicted probabilities were multiplied by 100 to obtain predicted percentages. Sensitivity analyses reexamined multivariable associations of year 1 alcohol intake by relaxing the p-value threshold for inclusion in the multivariable model to *p* < 0.2.

Change in categorical alcohol intake from baseline to year 1 was assessed using the McNemar test for dichotomized alcohol intake of < 1 drink per week vs. 1 or more and Bowker’s test of symmetry for the four-level alcohol intake measure. Following this, we examined bivariable associations of changes in alcohol intake from baseline to year 1 with characteristics of interest, again using Monte Carlo-based Fisher exact tests. For these associations, data were subsetted to participants with baseline alcohol intake falling in the middle two categories (1–4 and 5–14 drinks per week), since participants at the lower extreme of alcohol intake could not decrease alcohol consumption at year 1 and those at the higher extreme could not increase alcohol consumption at year 1. Change status was based on whether participants migrated from one of the four categorized alcohol intake categories (< 1, 1–4, 5–14, and 15 + drinks per week) at baseline to a different category at year 1. Participants moving one or more categories downward were classified as decreasing intake, those remaining in the same category were classified as no change, and those moving one or more categories upward were classified as increasing intake. Multivariable analyses again included all variables found to be bivariably significant (*p* < 0.05), and resulting marginal percentages calculated, similar to the year 1 cross-sectional analyses. Sensitivity analyses reexamined multivariable associations of change in alcohol intake from baseline to year 1 by relaxing the *p*-value threshold for inclusion in the multivariable model to *p* < 0.2.

All statistical tests were two-sided, and all analyses were carried out using the SAS system (SAS Institute, Inc., Cary, NC).

## Results

In all, 3252 patients with stage 0–3 breast cancer returned baseline questionnaires. Of these, we excluded 1022 patients who did not return the year 1 survey, one patient with disease recurrence prior to the year 1 survey, 112 patients with missing alcohol intake data on the baseline or year 1 survey, and 37 for whom time from diagnosis to questionnaire return was greater than 12 months, resulting in a final cohort of 2080 patients (Supplemental Fig. 1).

The mean age at diagnosis was 59.2 years (SD = 12.3), with 99.4% being female. The majority of participants were White (95.9%) and post-menopausal (78.4%). Education level was high, with 56.3% holding a Bachelor’s or graduate degree. Approximately one-fourth (24.7%) reported financial difficulty at the time of diagnosis. Regarding baseline lifestyle behaviors, 68.8% were never smokers, 70.3% reported no strenuous exercise, and 32.5% reported no moderate exercise per week. Approximately half had stage 1 tumors (49.3%) and lumpectomy (51.8%), and most had ER-positive disease (84.4%). Sixty-six percent received endocrine therapy, 31.2% received chemotherapy, and 57.2% received radiotherapy. Forty-eight patients (2.3%) had a pathologic germline *BRCA1/2* mutation. Full details on participant characteristics are provided in Supplemental Table 1.

### Cross-sectional associations of alcohol intake at year 1 with patient characteristics

Bivariable associations of alcohol intake at year 1 with demographic and clinical characteristics are provided in Supplemental Table 1. Participants who reported financial difficulty had a higher likelihood of consuming < 1 drink/week (67.6 vs. 57.5%, *p* < 0.001). Lower alcohol consumption was associated with lower educational attainment, such that < 1 alcoholic drink/week was reported by more patients with a high school diploma or less (69.3%, *p* = 0.006), and 1–4 drinks/week and 5–14 drinks/week were reported by more participants with higher education levels. Nearly four percent of current smokers reported consuming 15 + drinks/week, compared to 1.7% of former smokers and 0.4% of never smokers (*p* < 0.001). Compared to patients with higher levels of strenuous exercise, those reporting 0 min of strenuous exercise per week were more likely to abstain from alcohol (62.1% reported < 1 drink/week vs. 51%) and were also less likely to consume smaller amounts of alcohol (28.1% reported 1–4 drinks/week compared to 41.6%, *p* = 0.011). Patients reporting 0 min of moderate exercise per week were more likely to abstain from alcohol than those reporting high levels of moderate exercise of greater than 240 min (67.1% vs. 53.2%). Conversely, patients reporting greater than 240 min of moderate exercise per week were more likely to consume 15 + drinks/week than those reporting lower levels of moderate exercise (2% vs. 0.6%, *p* < 0.001). Compared to patients who underwent lumpectomy only, those who underwent mastectomy were more likely to abstain from alcohol intake (61.6% reported < 1 drink/week vs. 57.6%, *p* = 0.04). Those with stage III disease were more likely to have alcohol intake of either < 1 drink/week compared to those with stage 0 disease (71.8% vs. 54.7%) or > 15 drinks per week (1.8% vs. 0.3%, *p* = 0.002) alcohol intake. Patients who underwent chemotherapy were more likely to abstain from alcohol intake than those who did not undergo chemotherapy (67% vs. 57.1%, *p* < 0.001). Alcohol consumption was not associated with age at cancer diagnosis, gender, menopausal status, mild exercise, endocrine therapy or radiotherapy, *BRCA* mutation status, or breast cancer subtype (*p* > 0.05 for each).

Bivariable associations of alcohol intake at year 1 with PROMIS-10 health scores are reported in Supplemental Table 1. Patients with the lowest physical health T-scores were more likely to consume < 1 drink/week (70.7% vs. 54%) than those with higher scores. A similar association was seen between alcohol intake and the sum of the raw mental and physical health scores; however, alcohol intake was not significantly associated with mental health *T*-scores.

**Fig. 1:**
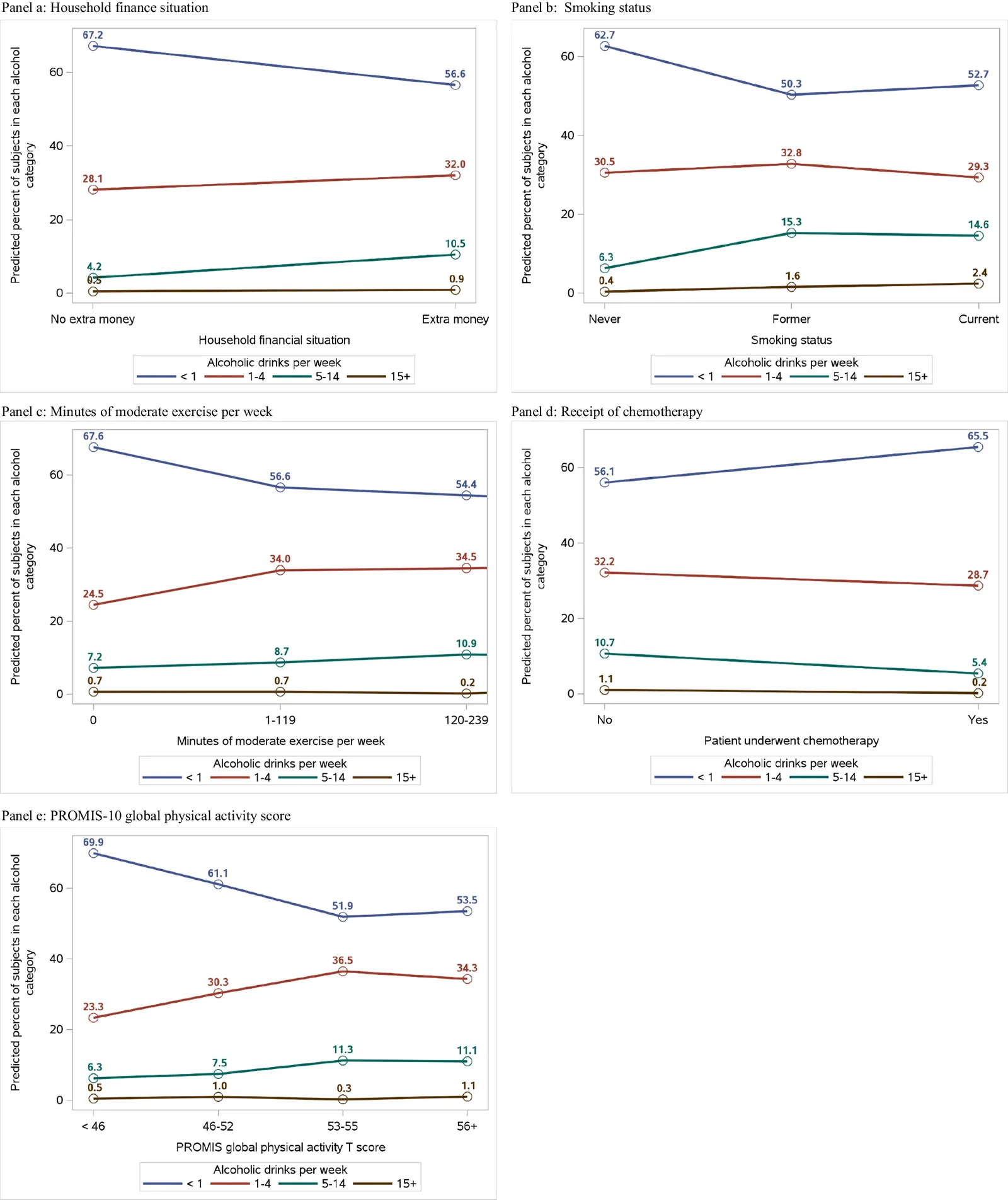
Effect plots of the variables that were significantly associated with alcohol intake at year 1 in multivariable models. Predicted percentages (*y*-axes) represent marginal predicted probabilities, calculated as the average percentage of individuals in each alcohol consumption category for the given demographic, behavioral, and clinical characteristics (*x*-axes) across all combinations of other variables in the multivariable model. **a** Household finance situation; **b** smoking status; **c** minutes of moderate exercise per week; **d** receipt of chemotherapy; **e** PROMIS-10 global physical activity score

In multivariable analyses, financial difficulty was again significantly associated with alcohol abstinence (67.2% of those with difficulty paying bills or little spare money for extra things reported < 1 drink/week compared to 56.6% for those with enough money for extra things after paying bills (*p* = 0.004, Table 1, Fig. 1a). In addition, patients who received chemotherapy (Fig. 1b) and those who never smoked (Fig. 1c) were more likely to abstain from alcohol than their demographic counterparts, similar to patterns observed in bivariable results. Multivariable-adjusted associations of alcohol with minutes of moderate exercise (Fig. 1d) and PROMIS-10 physical health T-scores (Fig. 1e) were also similar to bivariable results in that those reporting more exercise and higher physical health T-scores (> 56) were less likely to abstain from alcohol than those reporting fewer minutes of exercise and lower physical health T-scores. Education, strenuous exercise, tumor stage, and type of surgery were not associated with alcohol after adjustment for the other variables in the multivariable model (Table 1). Sensitivity analyses relaxing the bivariable p-value threshold for inclusion in the multivariable model to *p* < 0.2 revealed menopausal status to be associated with alcohol intake (*p* = 0.006 in the multivariable model). Compared to premenopausal women, postmenopausal women were more likely to abstain from alcohol (60.8% vs. 54.8%, Supplemental Fig. 2).

**Table 1:** Multivariable analysis of factors associated with year 1 alcohol intake

	Alcoholic drinks per week at year 1				
	< 1 (*N* = 1252)	1–4 (*N* = 630)	5–14 (*N* = 180)	15 + (*N* = 18)	*P*-value
Education (%)					0.093
High school graduate or less	65.0%	26.1%	7.6%	1.3%	
Vocational education beyond high school	68.9%	22.2%	7.4%	1.5%	
Some college or associate’s degree	60.2%	31.0%	7.4%	1.3%	
Bachelor’s degree	59.0%	31.8%	8.8%	0.4%	
Graduate school	53.5%	34.6%	11.5%	0.4%	
Household finance situation (%)					0.004
Difficulty paying bills or little spare money for extra things	67.2%	28.1%	4.2%	0.5%	
After paying bills, still enough money for extra things	56.6%	32.0%	10.5%	0.9%	
Smoking status at baseline (%)					< 0.001
Never	62.7%	30.5%	6.3%	0.4%	
Former	50.3%	32.8%	15.3%	1.6%	
Current	53.7%	29.3%	14.6%	2.4%	
Minutes of strenuous exercise per week at baseline (%)					0.158
0	61.6%	28.7%	8.9%	0.9%	
1–59	58.3%	30.6%	11.1%	0.0%	
60–119	55.2%	33.1%	10.3%	1.4%	
120 +	49.3%	42.5%	7.7%	0.5%	
Minutes of moderate exercise per week at baseline (%)					0.039
0	67.6%	24.5%	7.2%	0.7%	
1–119	56.6%	34.0%	8.7%	0.7%	
120–239	54.4%	34.5%	10.9%	0.2%	
240 +	52.2%	35.5%	10.2%	2.0%	
Tumor stage (%)					0.516
0	53.3%	36.2%	10.1%	0.4%	
1	57.6%	31.4%	10.1%	0.9%	
2	61.7%	30.0%	7.1%	1.2%	
3	71.6%	22.4%	6.0%	0.0%	
Type of surgery (%)					0.738
Lumpectomy only	56.8%	31.5%	10.9%	0.9%	
Mastectomy	61.4%	30.7%	7.1%	0.7%	
Patient underwent chemotherapy (%)					0.022
No	56.1%	32.2%	10.7%	1.1%	
Yes	65.5%	28.7%	5.4%	0.2%	
PROMIS global physical health T score, baseline (%)					0.036
< 46	69.9%	23.3%	6.3%	0.5%	
46–52	61.1%	30.3%	7.5%	1.0%	
53–55	51.9%	36.5%	11.3%	0.3%	
56 +	53.5%	34.3%	11.1%	1.1%

### Trend in alcohol intake from baseline to year 1

**Fig. 2:**
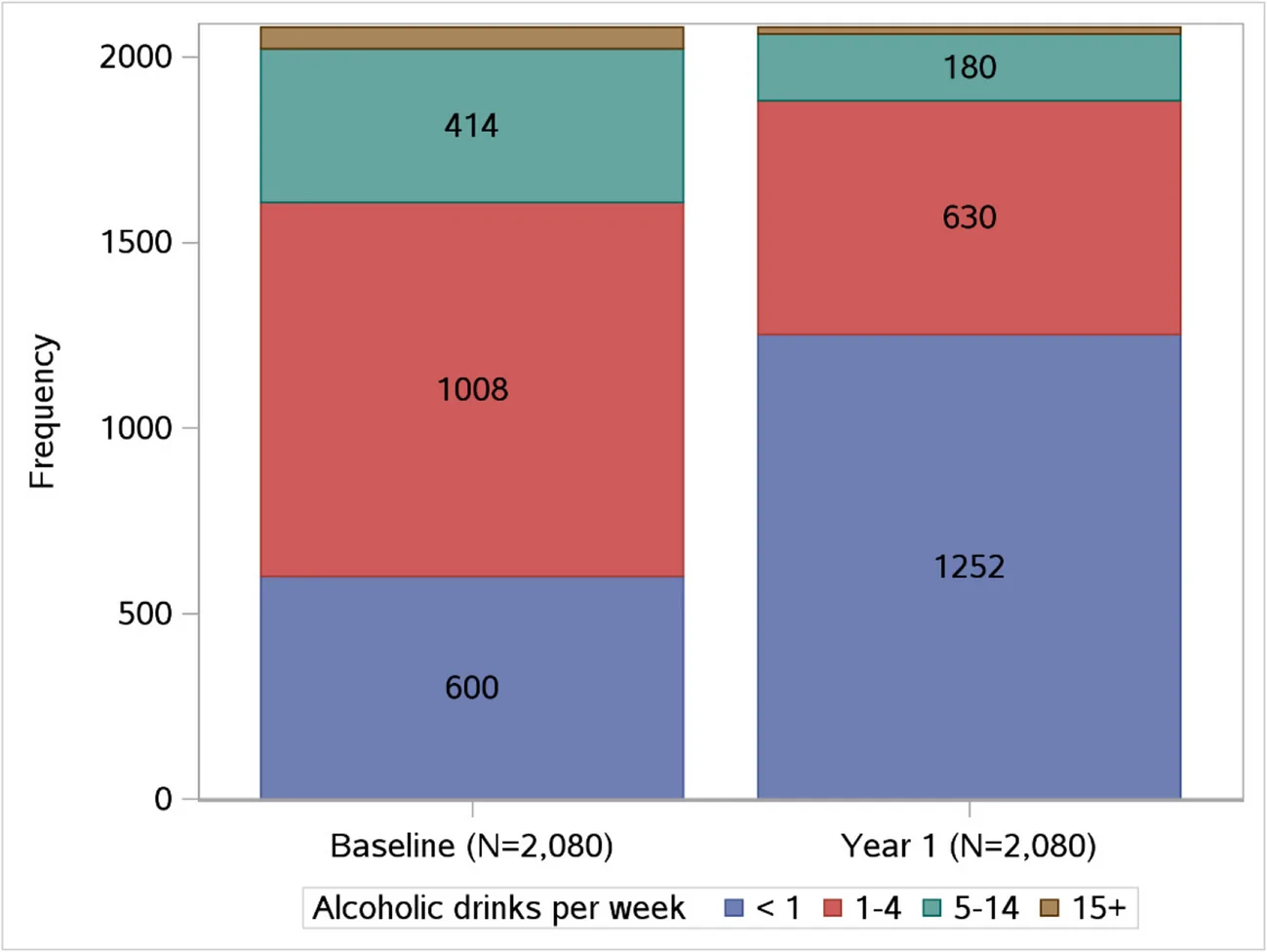
Self-reported categorical number of alcoholic drinks consumed per week, by survey year. Numbers are presented as a stacked bar chart. Baseline survey numbers are in the left-most bar, and year 1 survey numbers are in the right-most bar

At the year 1 follow-up, the proportion of participants drinking less than one drink per week increased to 60.2% (from 28.9% at baseline), while those consuming 1–4 drinks per week decreased to 30.3% (from 48.5%), and those drinking 5–14 drinks per week and 15 + drinks per week declined to 8.7% (from 19.9%) and 0.9% (from 2.8%), respectively (Supplemental Table 2, Fig. 2). Of those who reported drinking less than one drink per week at baseline, nearly all (98.5%) remained in this category at year 1. Among those who initially consumed 1–4 drinks per week, 39.2% remained in this category, while 59.1% reduced their intake to less than one drink per week, although a small proportion (1.7%) increased their consumption to five or more drinks per week. Participants who initially reported consuming 5–14 drinks exhibited notable declines in intake, with 51.7% reducing their consumption to 1–4 drinks per week and 13.5% decreasing to less than one drink per week. However, 33.3% of this group maintained their baseline intake, and 1.5% increased to 15 or more drinks per week. Similarly, among those who consumed 15 or more drinks per week at baseline, 43.1% reduced to 5–14 drinks, 20.7% reduced to 1–4 drinks, and 15.5% reduced to < 1 drink per week, while 20.7% maintained their high consumption.

Among participants who reported consuming at least one alcoholic drink per week at baseline, 55.3% (819/1480) continued this at year 1, while 44.7% (661/1480) reduced their intake to less than one drink per week. In contrast, among those who reported drinking less than one drink per week at baseline, the vast majority (98.5%, 591/600) remained in this category, with only 1.5% (9/600) increasing their consumption to one or more drinks per week. Overall, the proportion of participants consuming at least one drink per week decreased from 71.2% at baseline to 39.8% at year 1, while the proportion reporting less than one drink per week increased from 28.9 to 60.2%.

### Associations of change in alcohol intake from baseline to year 1 with patient characteristics

Bivariable associations of change in alcohol use from baseline to year 1 with demographic and clinical characteristics, among the 1422 participants not at the lowest or highest levels of baseline alcohol use, are presented in Supplemental Table 3. Participants who reported financial difficulty were more likely to decrease alcohol intake at year 1 (69.3% vs. 58.0%) than those who reported financial stability. Former smokers were less likely to reduce their alcohol intake (55.8%) compared to never smokers (63.4%) and current smokers (62.9%, *p* = 0.019). Compared to subjects not Engaging in moderate-intensity exercise, those Engaging in 240+ minutes of moderate-intensity exercise were less likely to reduce alcohol intake (51.7% vs. 65.1%, *p* = 0.021). A higher likelihood of a decrease in alcohol intake was seen in those with higher stage disease (stage III, 69.5% vs. stage 0 57.5%, *p* = 0.007) and those who received chemotherapy (70.6% vs. 56.5%, *p* < 0.001). A lower likelihood of a decrease in alcohol intake was observed in patients with estrogen receptor positive disease (59.2% vs. 68.4%, *p* = 0.027) and progesterone receptor positive disease (58.9% vs. 67.3%, *p* = 0.016), who were more likely to maintain stable alcohol intake than those with hormone-receptor negative disease.

Bivariable analysis of change in alcohol use by PROMIS-10 scores is reported in Supplemental Table 3. Participants with a physical health T score < 46 were more likely to reduce alcohol intake (69.4% vs. 55.5%, *p* < 0.001) compared to those with higher physical health T scores. A similar higher likelihood of a reduction in alcohol intake was seen in those with mental health T scores < 46 (67.1% vs. 55.2%, *p* = 0.008) and the lowest sum of raw mental and physical health scores (66.5% vs. 52.9%, *p* < 0.001).

**Fig. 3:**
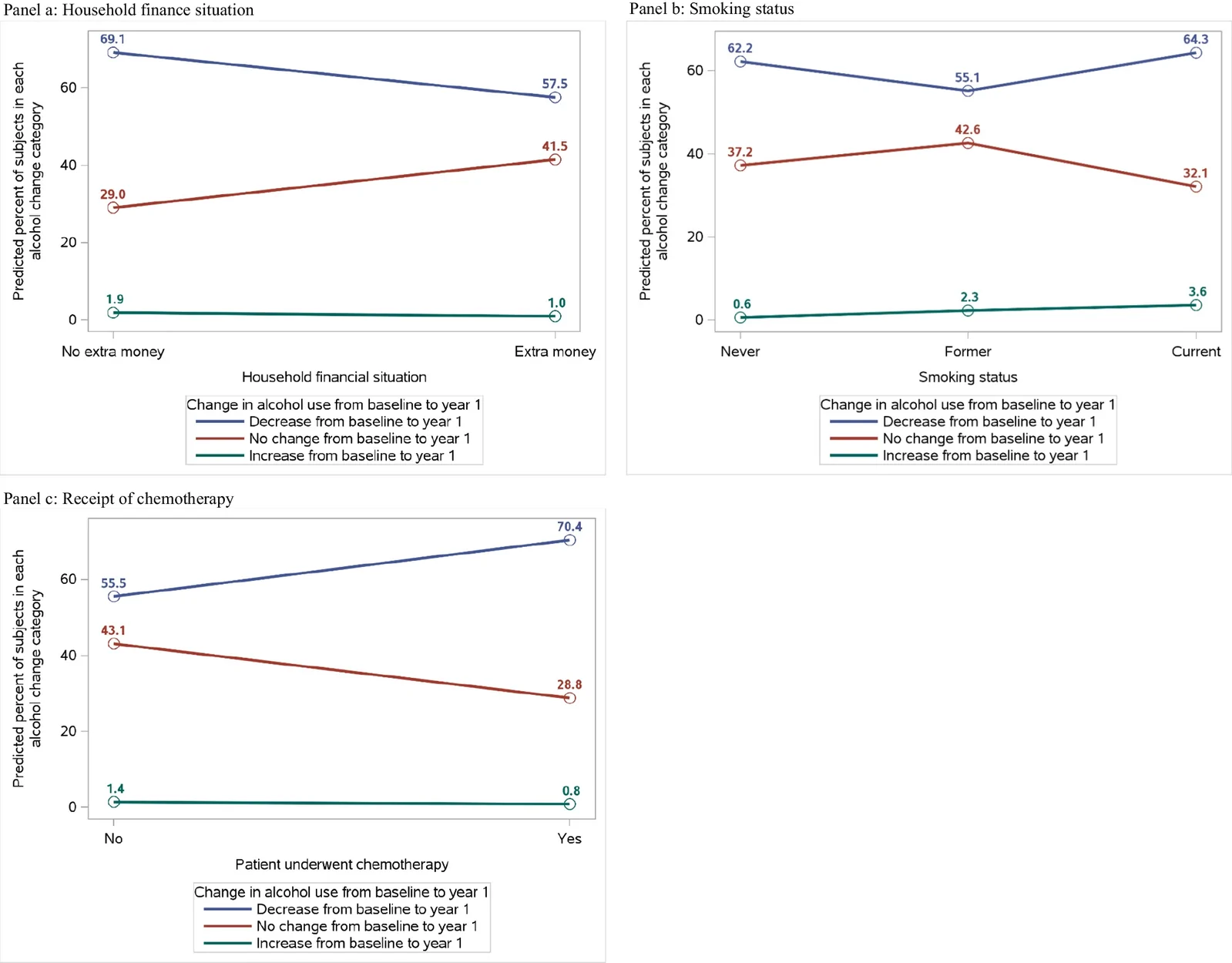
Effect plots of the variables that were significantly associated with change in alcohol intake from baseline to year 1 in multivariable models. Predicted percentages (*y*-axes) represent marginal predicted probabilities, calculated as the average percentage of individuals in each alcohol consumption category for the given demographic, behavioral, and clinical characteristics (*x*-axes) across all combinations of other variables in the multivariable model. **a** Household finance situation; **b** smoking status; **c** receipt of chemotherapy

In multivariable analyses, financial difficulty was again significantly associated with a decrease in alcohol use from baseline to year 1 (69.1% compared to 57.5% for those financially stable (Table 2, Fig. 3a)). Former smokers were less likely to reduce alcohol use (55.1%) compared to never (62.2%) and current smokers (64.3%) (Fig. 3b), and those who underwent chemotherapy were also more likely to reduce alcohol use (70.4%) than their demographic counterparts (55.5%) (Fig. 3c), similar to patterns observed in bivariable results. PROMIS-10 T scores, tumor stage, tumor characteristics, and type of surgery were not associated with change in alcohol intake from baseline to year 1 after adjustment for the other variables in the multivariable model (Supplemental Table 3). Sensitivity analyses relaxing the bivariable *p*-value threshold for inclusion in the multivariable model to *p* < 0.2 revealed moderate physical exercise to be associated with change in alcohol intake (*p* = 0.021 in the multivariable model). Participants reporting 120–239 min of moderate exercise per week were less likely to reduce their alcohol use compared to participants reporting less moderate physical exercise (Supplemental Fig. 3).

**Table 2:** Multivariable analysis of factors associated with change in alcohol from baseline to year 1

	Change in alcohol use from baseline to year 1			
	Decrease from baseline to year 1 (*N* = 866)	No change from baseline to year 1 (*N* = 533)	Increase from baseline to year 1 (*N* = 23)	*P*-value
Household finance situation (%)				0.022
Difficulty paying bills or little spare money for extra things	69.1%	29.0%	1.9%	
After paying bills, still enough money for extra things	57.5%	41.5%	1.0%	
Smoking status at baseline (%)				0.019
Never	62.2%	37.2%	0.6%	
Former	55.1%	42.6%	2.3%	
Current	64.3%	32.1%	3.6%	
Minutes of moderate exercise per week at baseline (%)				0.133
0	64.8%	33.9%	1.2%	
1–119	64.0%	34.7%	1.2%	
120–239	57.3%	41.5%	1.2%	
240 +	49.4%	49.4%	1.1%	
Tumor stage (%)				0.329
0	57.1%	41.3%	1.5%	
1	56.4%	42.8%	0.9%	
2	66.6%	32.5%	1.0%	
3	70.6%	25.9%	3.5%	
Type of surgery (%)				0.601
Lumpectomy only	56.9%	41.8%	1.3%	
Mastectomy	63.7%	35.2%	1.1%	
Patient underwent chemotherapy (%)				0.019
No	55.5%	43.1%	1.4%	
Yes	70.4%	28.8%	0.8%	
ER status of first BC (%)				0.611
Negative	68.3%	31.2%	0.5%	
Positive	58.5%	40.2%	1.3%	
PR status of first BC (%)				0.373
Negative	68.3%	30.6%	1.1%	
Positive	57.8%	41.0%	1.2%	
Categorized PROMIS global mental health T score, baseline (%)				0.451
< 46	63.0%	34.5%	2.6%	
46–52	64.3%	34.7%	1.0%	
53–55	63.2%	36.3%	0.5%	
56 +	54.3%	44.7%	0.9%	
PROMIS global physical health T score, baseline (%)				0.313
< 46	68.1%	29.8%	2.1%	
46–52	64.2%	34.6%	1.2%	
53–55	55.5%	44.1%	0.4%	
56 +	54.1%	44.7%	1.1%	

## Discussion

This study examined patterns of alcohol intake among breast cancer survivors in early survivorship. In a sample of 2080 patients, we identified key demographic, lifestyle, and clinical predictors of alcohol intake at one year post-diagnosis and changes in alcohol intake from baseline.

Financial difficulty emerged as a significant predictor of lower alcohol intake in early survivorship, with individuals reporting difficulty paying bills more likely to abstain from alcohol compared to those with financial stability. In addition, those experiencing financial hardship were more likely to reduce alcohol intake from baseline to year 1. These associations are consistent with general population studies, likely due to financial constraints and reduced social drinking opportunities for those who are struggling to pay their bills [24]. Higher income has been associated with moderate to high levels of alcohol intake, including higher likelihood of binge drinking, in other studies [25]. However, prior research has highlighted the complex relationship between financial distress and alcohol intake. Financial distress in cancer survivors has been linked to heightened psychological stress and depressive symptoms, causing some individuals to reduce alcohol intake due to concerns about exacerbating mental health symptoms, while others may increase consumption as a maladaptive coping strategy [26]. The literature suggests that individuals facing economic hardship may be at risk for unhealthy drinking patterns when they do consume alcohol, necessitating further exploration of drinking behaviors in this subgroup [27].

Our findings also revealed that smoking status was strongly associated with alcohol intake at year 1, as well as with change in alcohol use, with current smokers more likely to consume greater quantities of alcohol than former or never smokers at year 1. This is consistent with established behavioral clustering of smoking and alcohol intake, given overlapping psychosocial and behavioral risk factors due to shared neurobiological pathways involving reward processing, as well as common social and environmental influences. Unfortunately, prior studies have demonstrated that smoking and alcohol use may be associated with lower health consciousness, reduced adherence to medical recommendations, and greater engagement in other high-risk behaviors [28, 29]. In our study, we also found that former smokers were less likely to decrease their alcohol use from baseline to year 1 compared to current or never smokers. Compared to current smokers, former smokers may not perceive the same immediate health risks from alcohol as current smokers, who might be more motivated to change their behavior due to concerns about the compounded risks of ongoing smoking and alcohol use [30].

Exercise levels and physical health status were also associated with alcohol intake in early survivorship. Patients engaging in more moderate exercise were more likely to report alcohol consumption and less likely to reduce alcohol intake, a finding that has been reported in cancer survivor and general population studies. This may reflect a ‘compensatory health behavior’ phenomenon, wherein individuals engaging in beneficial health behaviors such as exercise perceive this to balance out the detrimental effects of alcohol intake [31–33]. Additionally, higher physical health T scores on the PROMIS-10 were associated with a greater likelihood of alcohol consumption, suggesting that better overall well-being may contribute to continued alcohol intake, perhaps as a function of culture and social life, with healthier individuals partaking in social drinking [34]. Engaging in regular exercise and experiencing a sense of well-being may therefore lead to misguided complacency in these individuals regarding their ongoing risk of cancer, and their health in general. Conversely, those with lower physical health scores were more likely to abstain from alcohol, which could be due to treatment-related side effects, poorer health, or avoidance of alcohol due to medication interactions [35].

Similarly, chemotherapy was strongly associated with a reduction in alcohol intake, possibly because chemotherapy can cause a metallic taste and reduce the pleasure associated with consuming certain drinks. Additionally, alcohol consumption may exacerbate common treatment-related side effects such as nausea, fatigue, and altered taste perception, further discouraging its use during treatment [36, 37]. The emphasis on supportive care and patient education during chemotherapy may also contribute to decreased alcohol intake, as patients are often counseled on lifestyle modifications to optimize treatment outcomes and minimize adverse effects [38].

Our study also found that postmenopausal breast cancer survivors were more likely than premenopausal women to abstain from alcohol use during early survivorship. Previous studies have demonstrated that postmenopausal women face a higher risk of breast cancer recurrence with increased alcohol intake [10, 14]. This may explain why postmenopausal survivors would be more likely to abstain or reduce alcohol consumption after diagnosis, potentially due to heightened awareness of recurrence risk and stronger adherence to guideline recommendations. Additionally, increased discomfort from vasomotor symptoms in postmenopausal women has previously been shown to be associated with reduced odds of drinking, perhaps because alcohol triggers hot flashes for some women [39].

A notable finding in our study was the significant decline in alcohol intake from baseline to year 1, with the odds of consuming ≥ 1 drink per week at year 1 markedly lower than at baseline. Nearly half of those who drank at least one drink per week prior to their diagnosis reduced their intake by one year after their diagnosis. This substantial decline is consistent with prior research indicating that a cancer diagnosis often prompts behavioral changes, including reductions in alcohol intake, due to heightened health awareness and provider counseling [35, 40]. Qualitative survivorship studies have reported that patients reduced or completely stopped drinking during treatment due to the side effects of therapy and an increased focus on their overall well-being [35]. This shift in behavior aligns with the concept of a cancer diagnosis serving as a “teachable moment”—a period when individuals may be more motivated to adopt healthier lifestyles, with a large proportion of patients expressing a desire to change their drinking behaviors [41].

Our findings have important implications for survivorship care and interventions aimed at reducing alcohol intake among breast cancer survivors. The observed declines in alcohol use over the first year post-diagnosis support the idea that the period immediately following a cancer diagnosis may be an important time for patients to receive alcohol-related risk education [21]. Our findings highlight the need for targeted alcohol intake interventions among specific high-risk behavioral subgroups, including smokers, those who are financially secure, and those reporting higher levels of exercise and overall well-being. Routine survivorship visits should incorporate counseling on alcohol’s impact on breast cancer outcomes, with particular attention to patients who may be less likely to modify their drinking behaviors without additional support, including those in better physical health (who may not perceive themselves to be at risk). Counseling that focuses on risk perception and utilizes motivational interviewing to address patients’ ambivalence toward alcohol intake may be helpful. Clinicians should consider following the SBIRT approach, which incorporates use of validated screening tools like the AUDIT-C into routine survivorship visits, brief conversations to increase awareness of unhealthy alcohol use, as well as referrals to treatment for those who screen positive for alcohol use disorder [42–44]. Behavioral counseling approaches that combine smoking cessation and alcohol reduction, as well as group-based survivorship programs with a focus on lifestyle management, may provide an avenue where patients feel supported in pursuing risk-reducing behaviors. Incorporating these efforts into the electronic medical record through survivorship care templates, nursing protocols, and primary care transition programs, along with equipping care teams with referral pathways to behavioral health specialists, should be considered [45].

Major strengths of our study are its large size and collection of a wide range of demographic, clinical, and behavioral data. Limitations include the fact that alcohol intake was self-reported, which may introduce recall and social desirability bias, potentially leading to underestimation of alcohol consumption. In addition, our cohort was predominantly White and highly educated, which may limit the generalizability of our findings to other populations. Prior research suggests that alcohol use patterns differ across racial and ethnic groups. White survivors have been found to have the highest likelihood of current alcohol consumption, and among those who currently drink, Hispanic survivors have been found to be more likely than non-Hispanic White survivors to engage in risky drinking patterns such as exceeding moderate limits or binge drinking [18, 25]. Our cohort lacks the racial and ethnic diversity to assess these patterns. Furthermore, while we identified several predictors of alcohol intake and changes over time, we were unable to explore the underlying psychological or social mechanisms driving these behaviors. Future research should investigate motivations for drinking or abstaining, perceived risks and benefits, and the role of social support in influencing alcohol intake patterns in breast cancer survivors.

In summary, our study provides important insights into patterns of alcohol intake among breast cancer survivors in early survivorship, identifying key demographic, clinical, and lifestyle factors associated with alcohol intake at 1 year after diagnosis and changes in alcohol intake from diagnosis to 1 year after diagnosis. Our findings underscore the importance of targeted interventions to support reductions in alcohol intake, particularly among more evidently high-risk groups such as current smokers, as well as those reporting high levels of financial stability and well-being, engaging in higher levels of exercise, and those for whom chemotherapy was not recommended. They also highlight the need for sustained alcohol-related counseling in survivorship care. As alcohol intake remains a modifiable factor with implications for breast cancer outcomes and overall health, future research should focus on developing and implementing effective strategies to promote alcohol reduction and adherence to survivorship guidelines.

Analyses were carried out using a multivariable multinomial logistic regression model using a glogit link function, fitting year 1 alcohol consumption as the outcome variable and simultaneously including all variables listed in the model as predictor variables. Row percents in the table, for a given level of a given characteristic, are marginal predicted percentages, averaging alcohol consumption percentages for that level across all combinations of all other characteristics in the model. *P* < 0.05 was considered statistically significant.

Analyses were carried out using a multivariable multinomial logistic regression model using a glogit link function, fitting change in alcohol consumption from baseline to year 1 as the outcome variable and simultaneously including all variables listed in the model as predictor variables. Row percents in the table, for a given level of a given characteristic are marginal predicted percentages, averaging alcohol consumption percentages for that level across all combinations of all other characteristics in the model. *P* < 0.05 was considered statistically significant.

## Supplementary Information

Below is the link to the electronic supplementary material.

Supplementary file1 (DOCX 326 KB)

## Data Availability

No datasets were generated or analyzed during the current study.
