# Supplementary material for: Factors associated with and patterns of alcohol intake during the first year of breast cancer survivorship
Source: J Cancer Surviv. 2025 Sep 24;20(5):2029–41. doi: 10.1007/s11764-025-01893-3 (PMC13723488; doi:10.1007/s11764-025-01893-3)
Supplement: Supplementary file 1 [file 11764_2025_1893_MOESM1_ESM.docx]

**Article Title:** Factors associated with and patterns of alcohol intake in early survivorship for breast cancer survivors

**Journal Name:** Journal of Cancer Survivorship

**Author names:** Sanjna Rajput, M.D.^1^, Robert A. Vierkant, M.S.^2^, Nicole L. Larson, M.P.H.^2^, Daniela L. Stan, M.D.^3^, Dawn M. Mussallem, D.O.^4^, Stacy D. D’Andre, M.D.^5^, Fergus J. Couch, Ph.D.^6^, Janet E. Olson, Ph.D.^2^, Ciara C. O’Sullivan, M.B., B.Ch. B.A.O.^5^, Kathryn J. Ruddy, M.D., M.P.H.^5^

**Affiliations:**

1. Mayo Clinic School of Graduate Medical Education, Internal Medicine Residency, Mayo Clinic, Rochester, Minnesota

2. Department of Quantitative Health Sciences, Mayo Clinic, Rochester, Minnesota

3. Department of General Internal Medicine, Mayo Clinic, Rochester, Minnesota

4. Department of General Internal Medicine, Mayo Clinic, Jacksonville, Florida

5. Department of Oncology, Mayo Clinic, Rochester, Minnesota

6. Department of Laboratory Medicine and Pathology Mayo Clinic, Rochester, Minnesota

**Corresponding author information:** Address correspondence to: Kathryn J. Ruddy, M.D. M.P.H., 200 First Street SW, Rochester, Minnesota 55905, [ruddy.kathryn@mayo.edu](mailto:ruddy.kathryn@mayo.edu)

Supplemental Table 1: Univariable associations of patient characteristics with Year 1 alcohol intake

|  | | | | | | |
| --- | --- | --- | --- | --- | --- | --- |
|  | Alcoholic drinks per week at Year 1 | | | |  | |
|  | < 1 (N=1252) | 1-4 (N=630) | 5-14 (N=180) | 15+ (N=18) | Total (N=2080) | P-value^1^ |
| **Gender**, n (%) |  |  |  |  |  | 0.412 |
| Female | 1246 (60.3%) | 625 (30.2%) | 178 (8.6%) | 18 (0.9%) | 2067 (99.4%) |  |
|  |  |  |  |  |  |  |
| **Age at cancer diagnosis**, n (%) |  |  |  |  |  | 0.129 |
| < 40 | 91 (58.0%) | 55 (35.0%) | 10 (6.4%) | 1 (0.6%) | 157 (7.5%) |  |
| 40-49 | 217 (61.0%) | 113 (31.7%) | 23 (6.5%) | 3 (0.8%) | 356 (17.1%) |  |
| 50-59 | 317 (58.3%) | 178 (32.7%) | 41 (7.5%) | 8 (1.5%) | 544 (26.2%) |  |
| 60-69 | 367 (62.4%) | 162 (27.6%) | 55 (9.4%) | 4 (0.7%) | 588 (28.3%) |  |
| 70+ | 260 (59.8%) | 122 (28.0%) | 51 (11.7%) | 2 (0.5%) | 435 (20.9%) |  |
|  |  |  |  |  |  |  |
| **Race**, n (%) |  |  |  |  |  | 0.158 |
| White | 1191 (59.7%) | 611 (30.6%) | 175 (8.8%) | 17 (0.9%) | 1994 (95.9%) |  |
|  |  |  |  |  |  |  |
| **Education**, n (%) |  |  |  |  |  | 0.006 |
| High school graduate or less | 165 (69.3%) | 54 (22.7%) | 16 (6.7%) | 3 (1.3%) | 238 (11.5%) |  |
| Vocational education beyond high school | 124 (67.0%) | 47 (25.4%) | 12 (6.5%) | 2 (1.1%) | 185 (8.9%) |  |
| Some college or associate’s degree | 294 (61.1%) | 145 (30.1%) | 35 (7.3%) | 7 (1.5%) | 481 (23.3%) |  |
| Bachelor's degree | 353 (58.5%) | 189 (31.3%) | 59 (9.8%) | 2 (0.3%) | 603 (29.2%) |  |
| Graduate school | 309 (55.1%) | 192 (34.2%) | 56 (10.0%) | 4 (0.7%) | 561 (27.1%) |  |
| Missing | 7 | 3 | 2 | 0 | 12 |  |
|  |  |  |  |  |  |  |
| **Household finance situation**, n (%) |  |  |  |  |  | <0.001 |
| Difficulty paying bills or little spare money for extra things | 345 (67.6%) | 135 (26.5%) | 27 (5.3%) | 3 (0.6%) | 510 (24.7%) |  |
| After paying bills, still enough money for extra things | 892 (57.5%) | 491 (31.7%) | 153 (9.9%) | 15 (1.0%) | 1551 (75.3%) |  |
| Missing | 15 | 4 | 0 | 0 | 19 |  |
|  |  |  |  |  |  |  |
| **Menopausal status**, n (%) |  |  |  |  |  | 0.079 |
| Pre-menopausal | 225 (55.3%) | 140 (34.4%) | 38 (9.3%) | 4 (1.0%) | 407 (21.6%) |  |
| Post-menopausal | 913 (61.9%) | 424 (28.7%) | 129 (8.7%) | 9 (0.6%) | 1475 (78.4%) |  |
| Missing | 114 | 66 | 13 | 5 | 198 |  |
|  |  |  |  |  |  |  |
| **Smoking status at Baseline**, n (%) |  |  |  |  |  | <0.001 |
| Never | 910 (64.4%) | 415 (29.3%) | 84 (5.9%) | 5 (0.4%) | 1414 (68.8%) |  |
| Former | 303 (51.4%) | 190 (32.2%) | 87 (14.7%) | 10 (1.7%) | 590 (28.7%) |  |
| Current | 29 (55.8%) | 14 (26.9%) | 7 (13.5%) | 2 (3.8%) | 52 (2.5%) |  |
| Missing | 10 | 11 | 2 | 1 | 24 |  |
|  |  |  |  |  |  |  |
| **Minutes of strenuous exercise per week at Baseline**, n (%) |  |  |  |  |  | 0.011 |
| 0 | 774 (62.1%) | 350 (28.1%) | 111 (8.9%) | 11 (0.9%) | 1246 (70.3%) |  |
| 1-59 | 72 (60.0%) | 36 (30.0%) | 12 (10.0%) | 0 (0.0%) | 120 (6.8%) |  |
| 60-119 | 88 (54.0%) | 57 (35.0%) | 16 (9.8%) | 2 (1.2%) | 163 (9.2%) |  |
| 120+ | 124 (51.0%) | 101 (41.6%) | 17 (7.0%) | 1 (0.4%) | 243 (13.7%) |  |
| Missing | 194 | 86 | 24 | 4 | 308 |  |
|  |  |  |  |  |  |  |
| **Minutes of moderate exercise per week at Baseline**, n (%) |  |  |  |  |  | <0.001 |
| 0 | 408 (67.1%) | 153 (25.2%) | 42 (6.9%) | 5 (0.8%) | 608 (32.5%) |  |
| 1-119 | 273 (57.7%) | 157 (33.2%) | 40 (8.5%) | 3 (0.6%) | 473 (25.3%) |  |
| 120-239 | 272 (54.8%) | 171 (34.5%) | 52 (10.5%) | 1 (0.2%) | 496 (26.5%) |  |
| 240+ | 156 (53.2%) | 104 (35.5%) | 27 (9.2%) | 6 (2.0%) | 293 (15.7%) |  |
| Missing | 143 | 45 | 19 | 3 | 210 |  |
|  |  |  |  |  |  |  |
| **Minutes of mild exercise per week at Baseline**, n (%) |  |  |  |  |  | 0.183 |
| 0 | 250 (64.3%) | 108 (27.8%) | 27 (6.9%) | 4 (1.0%) | 389 (19.9%) |  |
| 1-119 | 359 (58.3%) | 191 (31.0%) | 63 (10.2%) | 3 (0.5%) | 616 (31.6%) |  |
| 120-239 | 301 (57.0%) | 171 (32.4%) | 52 (9.8%) | 4 (0.8%) | 528 (27.1%) |  |
| 240+ | 253 (60.5%) | 131 (31.3%) | 28 (6.7%) | 6 (1.4%) | 418 (21.4%) |  |
| Missing | 89 | 29 | 10 | 1 | 129 |  |
|  |  |  |  |  |  |  |
| **Tumor stage**, n (%) |  |  |  |  |  | 0.002 |
| 0 | 182 (54.7%) | 120 (36.0%) | 30 (9.0%) | 1 (0.3%) | 333 (16.1%) |  |
| 1 | 601 (58.8%) | 312 (30.5%) | 101 (9.9%) | 8 (0.8%) | 1022 (49.3%) |  |
| 2 | 343 (62.6%) | 160 (29.2%) | 39 (7.1%) | 6 (1.1%) | 548 (26.4%) |  |
| 3 | 122 (71.8%) | 37 (21.8%) | 8 (4.7%) | 3 (1.8%) | 170 (8.2%) |  |
| Missing | 4 | 1 | 2 | 0 | 7 |  |
|  |  |  |  |  |  |  |
| **Type of surgery**, n (%) |  |  |  |  |  | 0.040 |
| Lumpectomy only | 594 (57.6%) | 322 (31.2%) | 107 (10.4%) | 9 (0.9%) | 1032 (51.8%) |  |
| Mastectomy | 592 (61.6%) | 293 (30.5%) | 67 (7.0%) | 9 (0.9%) | 961 (48.2%) |  |
| Missing | 66 | 15 | 6 | 0 | 87 |  |
|  |  |  |  |  |  |  |
| **Patient underwent endocrine therapy**, n (%) |  |  |  |  |  | 0.537 |
| Yes | 825 (59.6%) | 432 (31.2%) | 115 (8.3%) | 13 (0.9%) | 1385 (66.6%) |  |
|  |  |  |  |  |  |  |
| **Patient underwent chemotherapy**, n (%) |  |  |  |  |  | <0.001 |
| Yes | 435 (67.0%) | 173 (26.7%) | 36 (5.5%) | 5 (0.8%) | 649 (31.2%) |  |
|  |  |  |  |  |  |  |
| **Patient underwent radiotherapy**, n (%) |  |  |  |  |  | 0.850 |
| Yes | 716 (60.2%) | 361 (30.4%) | 100 (8.4%) | 12 (1.0%) | 1189 (57.2%) |  |
|  |  |  |  |  |  |  |
| **Pathologic BRCA1/2 mutation**, n (%) |  |  |  |  |  | 0.610 |
| Yes | 25 (52.1%) | 18 (37.5%) | 5 (10.4%) | 0 (0.0%) | 48 (2.3%) |  |
|  |  |  |  |  |  |  |
| **ER status of first BC**, n (%) |  |  |  |  |  | 0.575 |
| Negative | 199 (63.0%) | 93 (29.4%) | 23 (7.3%) | 1 (0.3%) | 316 (15.6%) |  |
| Positive | 1017 (59.7%) | 521 (30.6%) | 151 (8.9%) | 15 (0.9%) | 1704 (84.4%) |  |
| Missing | 36 | 16 | 6 | 2 | 60 |  |
|  |  |  |  |  |  |  |
| **PR status of first BC**, n (%) |  |  |  |  |  | 0.175 |
| Negative | 289 (64.2%) | 129 (28.7%) | 29 (6.4%) | 3 (0.7%) | 450 (22.4%) |  |
| Positive | 925 (59.3%) | 479 (30.7%) | 143 (9.2%) | 12 (0.8%) | 1559 (77.6%) |  |
| Missing | 38 | 22 | 8 | 3 | 71 |  |
|  |  |  |  |  |  |  |
| **HER2 status of first BC**, n (%) |  |  |  |  |  | 0.671 |
| Negative | 889 (61.1%) | 431 (29.6%) | 124 (8.5%) | 12 (0.8%) | 1456 (87.8%) |  |
| Positive | 127 (62.9%) | 56 (27.7%) | 16 (7.9%) | 3 (1.5%) | 202 (12.2%) |  |
| Missing | 236 | 143 | 40 | 3 | 422 |  |
|  |  |  |  |  |  |  |
|  |  |  |  |  |  |  |
| **Categorized PROMIS global mental health T score, Baseline**, n (%) |  |  |  |  |  | 0.063 |
| < 46 | 317 (66.2%) | 122 (25.5%) | 37 (7.7%) | 3 (0.6%) | 479 (23.1%) |  |
| 46-52 | 307 (60.9%) | 148 (29.4%) | 43 (8.5%) | 6 (1.2%) | 504 (24.3%) |  |
| 53-55 | 219 (60.5%) | 115 (31.8%) | 26 (7.2%) | 2 (0.6%) | 362 (17.5%) |  |
| 56+ | 406 (55.7%) | 243 (33.3%) | 73 (10.0%) | 7 (1.0%) | 729 (35.1%) |  |
| Missing | 3 | 2 | 1 | 0 | 6 |  |
|  |  |  |  |  |  |  |
| **PROMIS global physical health T score, Baseline**, n (%) |  |  |  |  |  | <0.001 |
| < 46 | 353 (70.7%) | 110 (22.0%) | 34 (6.8%) | 2 (0.4%) | 499 (24.4%) |  |
| 46-52 | 368 (60.8%) | 186 (30.7%) | 44 (7.3%) | 7 (1.2%) | 605 (29.5%) |  |
| 53-55 | 216 (54.3%) | 141 (35.4%) | 40 (10.1%) | 1 (0.3%) | 398 (19.4%) |  |
| 56+ | 295 (54.0%) | 186 (34.1%) | 57 (10.4%) | 8 (1.5%) | 546 (26.7%) |  |
| Missing | 20 | 7 | 5 | 0 | 32 |  |
|  |  |  |  |  |  |  |
| **Categorized PROMIS 10 sum of raw mental and physical health scores, Baseline**, n (%) |  |  |  |  |  | 0.001 |
| < 30 | 414 (66.6%) | 159 (25.6%) | 46 (7.4%) | 3 (0.5%) | 622 (30.5%) |  |
| 30-32 | 321 (61.8%) | 154 (29.7%) | 38 (7.3%) | 6 (1.2%) | 519 (25.4%) |  |
| 33-34 | 228 (57.3%) | 135 (33.9%) | 32 (8.0%) | 3 (0.8%) | 398 (19.5%) |  |
| 35+ | 266 (52.9%) | 173 (34.4%) | 58 (11.5%) | 6 (1.2%) | 503 (24.6%) |  |
| Missing | 23 | 9 | 6 | 0 | 38 |  |
|  |  |  |  |  |  |  |
| 1. Fisher exact test. P<0.05 was considered statistically significant. | | | | | | |

N, number of participants; %, row percentages

Supplemental Table 2. Comparison of alcohol intake per week as reported in the Baseline survey to that reported in the Year 1 survey.

| Drinks per week, Baseline | Drinks per week, Year 1 | | | | |
| --- | --- | --- | --- | --- | --- |
|  | < 1 | 1-4 | 5-14 | 15+ | Total |
| < 1 | 591 | 9 | 0 | 0 | 600 (28.9%) |
| 1-4 | 596 | 395 | 17 | 0 | 1008 (48.5%) |
| 5-14 | 56 | 214 | 138 | 6 | 414 (19.9%) |
| 15+ | 9 | 12 | 25 | 12 | 58 (2.8%) |
| Total | 1252 (60.2%) | 630 (30.3%) | 180 (8.7%) | 18 (0.9%) | 2080 |

Supplemental Table 3: Univariable associations of change in alcohol use from Baseline to Year 1 with demographic and clinical characteristics

|  | | | | | |
| --- | --- | --- | --- | --- | --- |
|  | Change in alcohol use from baseline to Year 1 | | |  | |
|  | Decreased (N=866) | Unchanged (N=533) | Increased (N=23) | Total (N=1422) | P-value^1^ |
| **Gender**, n (%) |  |  |  |  | 0.197 |
| Female | 860 (60.9%) | 530 (37.5%) | 22 (1.6%) | 1412 (99.3%) |  |
|  |  |  |  |  |  |
| **Age at cancer diagnosis**, n (%) |  |  |  |  | 0.095 |
| < 40 | 83 (65.9%) | 38 (30.2%) | 5 (4.0%) | 126 (8.9%) |  |
| 40-49 | 167 (67.3%) | 78 (31.5%) | 3 (1.2%) | 248 (17.4%) |  |
| 50-59 | 227 (58.4%) | 156 (40.1%) | 6 (1.5%) | 389 (27.4%) |  |
| 60-69 | 222 (59.0%) | 148 (39.4%) | 6 (1.6%) | 376 (26.4%) |  |
| 70+ | 167 (59.0%) | 113 (39.9%) | 3 (1.1%) | 283 (19.9%) |  |
|  |  |  |  |  |  |
| **Race**, n (%) |  |  |  |  | 0.811 |
| White | 838 (60.7%) | 519 (37.6%) | 23 (1.7%) | 1380 (97.0%) |  |
|  |  |  |  |  |  |
| **Education**, n (%) |  |  |  |  | 0.239 |
| High school graduate or less | 87 (61.3%) | 54 (38.0%) | 1 (0.7%) | 142 (10.0%) |  |
| Vocational education beyond high school | 84 (68.3%) | 35 (28.5%) | 4 (3.3%) | 123 (8.7%) |  |
| Some college or associate’s degree | 208 (62.8%) | 118 (35.6%) | 5 (1.5%) | 331 (23.4%) |  |
| Bachelor's degree | 249 (59.6%) | 165 (39.5%) | 4 (1.0%) | 418 (29.5%) |  |
| Graduate school | 236 (58.7%) | 157 (39.1%) | 9 (2.2%) | 402 (28.4%) |  |
| Missing | 2 | 4 | 0 | 6 |  |
|  |  |  |  |  |  |
| **Household finance situation**, n (%) |  |  |  |  | <0.001 |
| Difficulty paying bills or little spare money for extra things | 233 (69.3%) | 96 (28.6%) | 7 (2.1%) | 336 (23.8%) |  |
| After paying bills, still enough money for extra things | 623 (58.0%) | 436 (40.6%) | 16 (1.5%) | 1075 (76.2%) |  |
| Missing | 10 | 1 | 0 | 11 |  |
|  |  |  |  |  |  |
| **Menopausal status**, n (%) |  |  |  |  | 0.924 |
| Pre-menopausal | 179 (59.7%) | 117 (39.0%) | 4 (1.3%) | 300 (23.3%) |  |
| Post-menopausal | 604 (61.2%) | 369 (37.4%) | 14 (1.4%) | 987 (76.7%) |  |
| Missing | 83 | 47 | 5 | 135 |  |
|  |  |  |  |  |  |
| **Smoking status at Baseline**, n (%) |  |  |  |  | 0.019 |
| Never | 595 (63.4%) | 333 (35.5%) | 10 (1.1%) | 938 (66.9%) |  |
| Former | 240 (55.8%) | 178 (41.4%) | 12 (2.8%) | 430 (30.6%) |  |
| Current | 22 (62.9%) | 12 (34.3%) | 1 (2.9%) | 35 (2.5%) |  |
| Missing | 9 | 10 | 0 | 19 |  |
|  |  |  |  |  |  |
| **Minutes of strenuous exercise per week at Baseline**, n (%) |  |  |  |  | 0.633 |
| 0 | 504 (61.8%) | 301 (36.9%) | 10 (1.2%) | 815 (66.7%) |  |
| 1-59 | 61 (64.9%) | 31 (33.0%) | 2 (2.1%) | 94 (7.7%) |  |
| 60-119 | 72 (57.6%) | 51 (40.8%) | 2 (1.6%) | 125 (10.2%) |  |
| 120+ | 107 (56.9%) | 78 (41.5%) | 3 (1.6%) | 188 (15.4%) |  |
| Missing | 122 | 72 | 6 | 200 |  |
|  |  |  |  |  |  |
| **Minutes of moderate exercise per week at Baseline**, n (%) |  |  |  |  | 0.021 |
| 0 | 246 (65.1%) | 125 (33.1%) | 7 (1.9%) | 378 (29.1%) |  |
| 1-119 | 228 (63.9%) | 125 (35.0%) | 4 (1.1%) | 357 (27.5%) |  |
| 120-239 | 208 (57.5%) | 149 (41.2%) | 5 (1.4%) | 362 (27.9%) |  |
| 240+ | 104 (51.7%) | 95 (47.3%) | 2 (1.0%) | 201 (15.5%) |  |
| Missing | 80 | 39 | 5 | 124 |  |
|  |  |  |  |  |  |
| **Minutes of mild exercise per week at Baseline**, n (%) |  |  |  |  | 0.673 |
| 0 | 140 (57.9%) | 97 (40.1%) | 5 (2.1%) | 242 (18.0%) |  |
| 1-119 | 269 (60.0%) | 173 (38.6%) | 6 (1.3%) | 448 (33.3%) |  |
| 120-239 | 220 (59.6%) | 144 (39.0%) | 5 (1.4%) | 369 (27.4%) |  |
| 240+ | 187 (65.2%) | 96 (33.4%) | 4 (1.4%) | 287 (21.3%) |  |
| Missing | 50 | 23 | 3 | 76 |  |
|  |  |  |  |  |  |
| **Tumor stage**, n (%) |  |  |  |  | 0.007 |
| 0 | 134 (57.5%) | 95 (40.8%) | 4 (1.7%) | 233 (16.4%) |  |
| 1 | 413 (58.4%) | 287 (40.6%) | 7 (1.0%) | 707 (49.9%) |  |
| 2 | 244 (65.4%) | 122 (32.7%) | 7 (1.9%) | 373 (26.3%) |  |
| 3 | 73 (69.5%) | 28 (26.7%) | 4 (3.8%) | 105 (7.4%) |  |
| Missing | 2 | 1 | 1 | 4 |  |
|  |  |  |  |  |  |
| **Type of surgery**, n (%) |  |  |  |  | 0.035 |
| Lumpectomy only | 409 (57.4%) | 293 (41.1%) | 11 (1.5%) | 713 (52.0%) |  |
| Mastectomy | 422 (64.1%) | 226 (34.3%) | 10 (1.5%) | 658 (48.0%) |  |
| Missing | 35 | 14 | 2 | 51 |  |
|  |  |  |  |  |  |
| **Patient underwent endocrine therapy**, n (%) |  |  |  |  | 0.172 |
| Yes | 572 (59.8%) | 372 (38.9%) | 13 (1.4%) | 957 (67.3%) |  |
|  |  |  |  |  |  |
| **Patient underwent chemotherapy**, n (%) |  |  |  |  | <0.001 |
| Yes | 314 (70.6%) | 124 (27.9%) | 7 (1.6%) | 445 (31.3%) |  |
|  |  |  |  |  |  |
| **Patient underwent radiotherapy**, n (%) |  |  |  |  | 0.955 |
| Yes | 499 (60.7%) | 309 (37.6%) | 14 (1.7%) | 822 (57.8%) |  |
|  |  |  |  |  |  |
| **Pathologic BRCA1/2 mutation**, n (%) |  |  |  |  | 0.604 |
| Yes | 22 (55.0%) | 18 (45.0%) | 0 (0.0%) | 40 (2.8%) |  |
|  |  |  |  |  |  |
| **ER status of first BC**, n (%) |  |  |  |  | 0.027 |
| Negative | 160 (68.4%) | 71 (30.3%) | 3 (1.3%) | 234 (17.0%) |  |
| Positive | 679 (59.2%) | 450 (39.3%) | 17 (1.5%) | 1146 (83.0%) |  |
| Missing | 27 | 12 | 3 | 42 |  |
|  |  |  |  |  |  |
| **PR status of first BC**, n (%) |  |  |  |  | 0.016 |
| Negative | 210 (67.3%) | 97 (31.1%) | 5 (1.6%) | 312 (22.8%) |  |
| Positive | 623 (58.9%) | 421 (39.8%) | 14 (1.3%) | 1058 (77.2%) |  |
| Missing | 33 | 15 | 4 | 52 |  |
|  |  |  |  |  |  |
| **HER2 status of first BC**, n (%) |  |  |  |  | 0.768 |
| Negative | 611 (61.6%) | 368 (37.1%) | 13 (1.3%) | 992 (88.0%) |  |
| Positive | 86 (63.7%) | 47 (34.8%) | 2 (1.5%) | 135 (12.0%) |  |
| Missing | 169 | 118 | 8 | 295 |  |
|  |  |  |  |  |  |
|  |  |  |  |  |  |
| **Categorized PROMIS global mental health T score, Baseline**, n (%) |  |  |  |  | 0.008 |
| < 46 | 210 (67.1%) | 95 (30.4%) | 8 (2.6%) | 313 (22.1%) |  |
| 46-52 | 213 (61.9%) | 126 (36.6%) | 5 (1.5%) | 344 (24.3%) |  |
| 53-55 | 160 (63.0%) | 92 (36.2%) | 2 (0.8%) | 254 (17.9%) |  |
| 56+ | 279 (55.2%) | 219 (43.4%) | 7 (1.4%) | 505 (35.7%) |  |
| Missing | 4 | 1 | 1 | 6 |  |
|  |  |  |  |  |  |
| **PROMIS global physical health T score, Baseline**, n (%) |  |  |  |  | <0.001 |
| < 46 | 211 (69.4%) | 86 (28.3%) | 7 (2.3%) | 304 (21.7%) |  |
| 46-52 | 267 (63.7%) | 144 (34.4%) | 8 (1.9%) | 419 (29.9%) |  |
| 53-55 | 159 (55.6%) | 124 (43.4%) | 3 (1.0%) | 286 (20.4%) |  |
| 56+ | 217 (55.5%) | 169 (43.2%) | 5 (1.3%) | 391 (27.9%) |  |
| Missing | 12 | 10 | 0 | 22 |  |
|  |  |  |  |  |  |
| **Categorized PROMIS 10 sum of raw mental and physical health scores, Baseline**, n (%) |  |  |  |  | <0.001 |
| < 30 | 266 (66.5%) | 125 (31.3%) | 9 (2.3%) | 400 (28.7%) |  |
| 30-32 | 235 (65.5%) | 119 (33.1%) | 5 (1.4%) | 359 (25.8%) |  |
| 33-34 | 160 (57.6%) | 114 (41.0%) | 4 (1.4%) | 278 (19.9%) |  |
| 35+ | 189 (52.9%) | 164 (45.9%) | 4 (1.1%) | 357 (25.6%) |  |
| Missing | 16 | 11 | 1 | 28 |  |
|  |  |  |  |  |  |
| 1. Fisher exact test. P<0.05 was considered statistically significant. | | | | | |

N, number of participants; %, row percentages

Supplemental Figure 1. CONSORT diagram of study exclusions

Baseline survey mailed to 4440 patients with Breast cancer

2117 had non-missing alcohol intake for both the Baseline and Year 1 survey

2229 participants were recurrence-free after one year post-breast cancer

2230 returned Year 1 survey

**Final sample size: N=2080**

37 excluded because time from diagnosis to return of Baseline survey was more than one year

112 had missing alcohol intake on Baseline or Year 1 survey

1 had disease recurrence within one year

1022 failed to return Year 1 Survey

3252 participants excluding Stage 4 breast cancer returned Baseline survey

Supplemental Figure 2: Effect plot of the association between Year 1 alcohol intake and menopausal status in sensitivity analysis multivariable model. Predicted percentages (Y axes) represent marginal predicted probabilities, calculated as the average percentage of individuals in each alcohol consumption category by levels of menopausal status (X axes) across all combinations of other variables in the multivariable model.

Supplemental Figure 3: Effect plot of the association between with change in alcohol intake from Baseline to Year 1 and number of weekly minutes of moderate exercise in sensitivity analysis multivariable model. Predicted percentages (Y axes) represent marginal predicted probabilities, calculated as the average percentage of individuals in each alcohol consumption change category by levels of moderate exercise (X axes) across all combinations of other variables in the multivariable model.
